# NMDA Receptors on Dopaminoceptive Neurons Are Essential for Drug-Induced Conditioned Place Preference^1^,^2^,^3^

**DOI:** 10.1523/ENEURO.0084-15.2016

**Published:** 2016-06-09

**Authors:** Magdalena Sikora, Krzysztof Tokarski, Bartosz Bobula, Joanna Zajdel, Kamila Jastrzębska, Przemysław Eligiusz Cieślak, Magdalena Zygmunt, Joanna Sowa, Magdalena Smutek, Katarzyna Kamińska, Krystyna Gołembiowska, David Engblom, Grzegorz Hess, Ryszard Przewlocki, Jan Rodriguez Parkitna

**Affiliations:** 1 Department of Molecular Neuropharmacology, Institute of Pharmacology, Polish Academy of Sciences, 31-343 Krakow, Poland; 2 Department of Physiology, Institute of Pharmacology, Polish Academy of Sciences, 31-343 Krakow, Poland; 3 Cell Biology, Department of Clinical and Experimental Medicine, Linköping University, SE-581 83 Linköping, Sweden; 4 Department of Pharmacology, Institute of Pharmacology, Polish Academy of Sciences, 31-343 Krakow, Poland; 5 Department of Neurobiology and Neuropsychology, Institute of Applied Psychology, Jagiellonian University, 30-348 Krakow, Poland

**Keywords:** dopamine, drugs of abuse, NMDA receptors, operant conditioning, reinforcement

## Abstract

Plasticity of the brain’s dopamine system plays a crucial role in adaptive behavior by regulating appetitive motivation and the control of reinforcement learning. In this study, we investigated drug- and natural-reward conditioned behaviors in a mouse model in which the NMDA receptor-dependent plasticity of dopaminoceptive neurons was disrupted. We generated a transgenic mouse line with inducible selective inactivation of the NR1 subunit in neurons expressing dopamine D1 receptors (the NR1^D1CreERT2^ mice). Whole-cell recordings of spontaneous EPSCs on neurons in the nucleus accumbens confirmed that a population of neurons lacked the NMDA receptor-dependent component of the current. This effect was accompanied by impaired long-term potentiation in the nucleus accumbens and in the CA1 area of the ventral, but not the dorsal, hippocampus. Mutant mice did not differ from control animals when tested for pavlovian or instrumental conditioning. However, NR1^D1CreERT2^ mice acquired no preference for a context associated with administration of drugs of abuse. In the conditioned place preference paradigm, mutant mice did not spend more time in the context paired with cocaine, morphine, or ethanol, although these mice acquired a preference for sucrose jelly and an aversion to naloxone injections, as normal. Thus, we observed that the selective inducible ablation of the NMDA receptors specifically blocks drug-associated context memory with no effect on positive reinforcement in general.

## Significance Statement

We find that removing the NMDA receptor, which is a regulator of plasticity in excitatory synapses, in a subpopulation of neurons receiving dopamine signals disrupted the rewarding effects of drugs of abuse within their environmental context. Simultaneously, the NMDA receptor loss had no effect on the memory of a context associated with a natural reward or with an aversive substance. This observation supports the hypothesis that neuronal mechanisms processing natural and drug rewards differ and shows that NMDA receptors in a subgroup of dopaminoceptive neurons are essential specifically in the latter case.

## Introduction

Dopamine neurons of the ventral midbrain form the core of the reward system that controls reinforcement learning and motivation (Wise, 2004; Salamone and Correa, 2012). Dopamine neurotransmission is involved in learning associations between cues and rewards and comparing outcomes with prior expectations (Schultz, 2015). On a neuronal level, dopamine acts on D1-like and D2-like metabotropic receptors, which are coupled to G_s_/G_olf_ and G_i_ proteins, respectively (Lachowicz and Sibley, 1997). The expression of D1- and D2-like receptors is particularly high in the striatum, including the nucleus accumbens (NAc), which is the main target area of midbrain dopamine neurons (Fremeau et al., 1991; Weiner et al., 1991). Almost all striatal neurons express either D1 or D2 dopamine receptors; however, few express both (Curran and Watson, 1995; Gerfen and Surmeier, 2011). D1 receptors are primarily present on the medium spiny neurons that project directly to the output nuclei of the basal ganglia (the direct pathway), whereas D2 receptors are located on the medium spiny neurons that do not project outside the globus pallidus/ventral pallidum (the indirect pathway). It should be noted that extent of overlap between D1 and D2 expression is probably highest in the NAc shell, where it was estimated that ∼17% of neurons express both receptors based on data from transgenic reporter mice (Bertran-Gonzalez et al., 2008). Dopamine receptors are also expressed in other areas of the forebrain, notably, the prefrontal cortex, amygdala, and hippocampus, where their expression is at least partly non-overlapping (Weiner et al., 1991; Hurd et al., 2001; Gangarossa et al., 2013).

Our understanding of the dopamine system plasticity underlying specific behavioral outcomes has been strongly influenced by research on addiction. Drugs of abuse, which have the ability to increase dopamine levels in the NAc, induce changes in AMPA/NMDA glutamate receptor ratios in the ventral tegmental area dopaminergic neurons, which persist for ∼10 days after a single drug exposure (Lüscher and Malenka, 2011). Similar adaptations are also triggered by natural rewards and by stress, and may be contingent on any stimulus that activates the dopamine system (Saal et al., 2003; Stuber et al., 2008). Changes in the activity of dopaminergic neurons lead to altered plasticity in the NAc (Mameli et al., 2009), where repeated drug exposure induces complex and dynamic changes in the efficacy of excitatory synapses (Thomas et al., 2001; Kourrich et al., 2007; Wolf, 2010; Pascoli et al., 2014). Further, altered synaptic plasticity in the NAc has been suggested to underlie compulsive drug-seeking (Kasanetz et al., 2010), incubation of craving (Conrad et al., 2008), and sensitivity to cue-induced reinstatement of cocaine seeking (McFarland and Kalivas, 2001; Gipson et al., 2013). Finally, a recent report using a combination of optogenetics and cell-type-specific transgene expression demonstrated that the synaptic plasticity of D1-expressing neurons of the NAc is required for cocaine seeking (Pascoli et al., 2014).

The research on addiction has not only revealed specific forms of neuronal plasticity associated with drug-induced pathological behaviors but also identified mechanisms that serve physiological functions under normal circumstances. The NMDA receptors play essential roles in synaptic plasticity necessary for striatum-dependent learning. Both NMDA receptor antagonist injection into the striatum and striatum-specific inactivation of NR1 or NR2B subunits disrupted action–outcome learning (Dang et al., 2006; Yin et al., 2008; Brigman et al., 2013). NMDA receptor antagonist injection into the NAc slows the acquisition of Pavlovian conditioning, which was specifically attributed to blocking the plasticity of D1-expressing neurons (Dalley et al., 2005; Yin et al., 2008). Accordingly, mutant mice with disrupted NMDA receptors in D1-positive neurons failed to acquire classical conditioning (Parker et al., 2011) and displayed attenuated drug-induced conditioned place preference (CPP) and psychomotor sensitization (Heusner and Palmiter, 2005; Beutler et al., 2011b). Collectively, these studies indicate that NMDA receptors on dopaminoceptive neurons are critical for dopamine-driven behaviors. However, the interpretation of many of the studies is limited by possible developmental effects of mutations and by a lack of selectivity for the D1 or D2 subpopulations. In this study, we present a new genetically modified mouse, the NR1^D1CreERT2^ strain, in which NR1 subunit inactivation can be selectively induced in neurons expressing dopamine D1 receptors in adult animals. Using this model, we investigated the contribution of NMDA receptor-dependent plasticity of excitatory synaptic inputs to D1-expressing neurons in reward-driven learning.

## Methods

### Animals

Behavioral procedures were approved by the Local Bioethics Committee and were conducted in accordance with local regulations. All strains were maintained as congenic with C57BL/6N. Behavioral and electrophysiological analyses were performed on male mice, both sexes were used in neurochemical experiments, and an additional cohort of female mice was tested for activity in the open-field apparatus. Mice were housed 2–5 per cage in rooms with a controlled temperature of 22±2°C under a 12 h light/dark cycle. Animals had *ad libitum* access to food (Labofeed H, WPiK) and water. Mice were treated with tamoxifen (Sigma-Aldrich) when they reached the age of 8–10 weeks. The treatment, which consisted of 10 intraperitoneal injections of tamoxifen dissolved in sunflower oil and filtered through a 0.22 µm membrane, was performed every ∼12 h for 5 d. The tamoxifen dose was 100 mg/kg at 5 µl/g. Mice were allowed to recuperate for at least 3 weeks before experiments commenced. Unless indicated otherwise, mice were killed in a CO_2_ chamber.

Control mice were tamoxifen-treated (0/0; flox/flox) animals and NR1^DATCreERT2^ were tamoxifen-treated (Tg/0; flox/flox) animals. In one case (Tg/0; flox/flox) mice with no tamoxifen treatment were used as an additional control (a “not induced” cohort).

There were three cases when the same cohort of mice was used in more than one experiment. The same group of mice was used in the T-maze, as well as pavlovian approach/conditioned reinforcement and a separate cohort of mice was first trained for food self-administration and then tested under progressive ratio and variable interval schedules. Another group of mice was tested for social interactions and then alcohol jelly preference.

Each of the CPP and conditioned place aversion (CPA) experiments was performed on a separate cohort of animals. The cocaine CPP is a pooled result from two cohorts. Separate cohorts were used for electrophysiology, biochemical, and molecular analyses.

### Genotyping

Genotyping of the D1CreERT2 transgene was performed on lysed tail tip biopsies using the primers GTGCAAGCTGAACAACAGGA and CCAGCATCCACATTCTCCTT, which targeted the iCre domain. Additionally, in the same reaction, CCATTTGCTGGAGTGACTCTG and TAAATCTGGCAAGCGAGACG primers were used as a positive control targeting the Dicer gene. Genotyping of the NR1 flox variant was performed using the primers GGACAGCCCCTGGAAGCAAAAT and GGACCAGGACTTGCAGTCCAAAT. Recombination of the NR1 gene was assessed using primers GGACAGCCCCTGGAAGCAAAAT (same as 5′ for genotyping) and CAGTGCCTGGTGCACACTTCC. The loxP sites are located at positions 25296380 and 25298871 on chromosome 2, which correspond to introns between exons 8–9, and 16–17, following the numbering from gene sequence ENSMUSG00000026959 and transcript ENSMUST00000028335 (GRCm38.p3).

### Immunofluorescence

Mice were perfused with 4% paraformaldehyde in PBS. Dissected brains were fixed in the same solution for 2 h at 4°C and then in 30% sucrose in PBS. Next, 40 µm free-floating sections were obtained on a cryostat. The sections were incubated in blocking solution (1% BSA and 0.3% Triton X-100 in PBS) and then incubated overnight at room temperature with the following primary antibodies: mouse anti-DARPP-32 (BD Biosciences, Catalog #611520; 1:1000) and rabbit anti-ppEnk (Neuromics, Catalog #14124; 1:1000). Subsequently, the sections were rinsed five times in PBS and incubated with the following secondary antibodies: donkey anti-mouse AlexaFluor 488 (1:1000) and donkey anti-rabbit AlexaFluor 647 (1:1000) for 2 h. Then, the sections were washed three times in PBS and mounted onto glass slides. No additional antibodies were used for tdTomato imaging.

### Tissue monoamine concentration measurements

Striatal tissue was homogenized in 0.1 m HClO_4_ and centrifuged at 10,000 × *g*, and the supernatant was filtered through a 0.22 μm membrane. Dopamine and metabolites (DOPAC and HVA) were analyzed by HPLC with coulochemical detection. Chromatography was performed using an Ultimate 3000 System (Dionex) and a Coulochem III coulochemical detector (model 5300, ESA) with a 5020 guard cell, a 5014B microdialysis cell and a Hypersil Gold-C18 analytical column (3 μm, 3 × 100 mm). The mobile phase was composed of 0.05 m potassium phosphate buffer adjusted to pH 3.9, 0.5 mm EDTA, 13 mg/l 1-octanesulfonic acid sodium salt, 3.1% methanol, and 0.93% acetonitrile. The flow rate during the analysis was 0.7 ml/min. The applied potential of a guard cell was +600 mV, whereas that of the microdialysis cell was E1 = −50 mV and E2 = + 300 mV. Sensitivity was set at 50 nA/V. The chromatographic data were processed using Chromeleon v6.80 software (Dionex).

### Whole-cell patch-clamp recordings in slices

NR^D1CreERT2^ mice (15–25 weeks old) were anesthetized with isoflurane (Aerrane, Baxter) and decapitated. The brains were removed and immersed in an ice-cold artificial CSF (ACSF) composed of the following (in mm):124 NaCl, 5 KCl, 2.5 CaCl_2_, 1.3 MgSO_4_, 1.25 KH_2_PO_4_, 24 NaHCO_3_, and 10 d-glucose, bubbled with 95% O_2_/5% CO_2_. Coronal slices (380 µm thick) were cut with a vibrating microtome (Leica) and incubated in ACSF bubbled with 95% O_2_/5% CO_2_ at 32°C for 4–8 h before measurements started. Then slices were placed in the recording chamber, which was mounted on a Zeiss Axioskop microscope (Zeiss), and superfused (3 ml/min) with Mg-free ACSF (32 ± 0.5°C) for 15 min. Patch pipettes had resistances ranging from 5 to 6 MΩ and were filled with internal solution (in mm: 122 K-gluconate, 5 NaCl, 2 MgCl_2_, 1 EGTA, 10 HEPES, 0.3 CaCl_2_, 5 NaATP, and 0.4 NaGTP; 290 mOsm, pH 7.2). Cells were randomly selected in the NAc core, 1.6–1.0 mm from the bregma. Only cells with a resting membrane potential of at least −50 mV and overshooting action potentials were used for analysis. The stimulus–response characteristics of recorded neurons were evaluated using rectangular current pulses (500 ms) of increasing intensity in 20 pA steps (60–600 pA). Then, cells were voltage-clamped at −76 mV, and spontaneous EPSCs (sEPSCs) were recorded for 4 min. To block NMDA receptor-mediated currents, the specific antagonist CGP37849 (10 mm) was added to the ACSF (Myme et al., 2003). After 15 min of perfusion, sEPSCs were recorded again for 4 min. Spontaneous EPSCs were detected off-line and analyzed using Mini Analysis software (Synaptosoft).

### Extracellular recordings in slices

Slices were prepared in the same manner as slices for the whole-cell patch-clamp procedure and then transferred to an interface-type recording chamber and perfused at 2 ml/min with ACSF (32 ± 0.3°C). Field potentials (FPs) were evoked by stimulation (0.033 Hz, duration 200 μs) using a constant-current stimulus isolation unit (WPI) and a bipolar Pt-Ir electrode (FHC). FPs were recorded from the NAc using ACSF-filled glass micropipettes (1–2 MΩ) placed in the NAc core, and the stimulating electrode was placed dorsally. Hippocampal field EPSPs (fEPSPs) were evoked by stimulating Schaffer collaterals and were recorded from the striatum radiatum of the CA1 area. Signals were amplified (Axoprobe 2, Axon Instruments), band-pass filtered (1 Hz–5 kHz), A/D converted (micro1401 interface, Signal 2 software, CED) and analyzed on-line and off-line. For long-term potentiation (LTP) induction, a high-frequency stimulation protocol (HFS) was used, with three trains of 100 pulses at 100 Hz and with a 3 min gap between trains. After HFS treatment, the stimulation intensity was adjusted to evoke a response of 30% of the maximum amplitude. In case of LTP in the hippocampus the slopes were used for calculations, however, in case of the NAc we used the amplitude. The reason to use amplitude in the case of NAc was low signal-to-noise ratio, which caused considerable variability in the slope values.

### Behavioral procedures

#### Drug-conditioned place preference

Cocaine, morphine, and naloxone were dissolved in sterile saline, ethanol was prepared as a 20% (v/v) solution in saline, all substances were administered intraperitoneally in a volume of 10 µl/g. The doses of ethanol (1.5g/kg), morphine (10 mg/kg), and naloxone (10 mg/kg) were selected based on previous reports (Skoubis et al., 2001; Rodriguez Parkitna et al., 2012; Rodriguez Parkitna et al., 2013). The dose of cocaine 25 mg/kg was reported to produce robust preference and no seizures (Piechota et al., 2010). The procedure was performed in three compartment place-preference boxes with auto-guillotine doors and lights (MED-CPP-MSAT, Med Associates). During the pre- and post-conditioning tests, mice were individually placed on the center grey compartment, from which the animal could freely explore all compartments for 20 min. Assignment of mice to the compartments was unbiased. During the conditioning days, mice were treated with either saline or drug (i.p.) immediately before placement in the appropriate compartment. For cocaine, morphine, and naloxone, the conditioning sessions lasted for 40 min; for ethanol, the session lasted 5 min. The number of pairings was three for cocaine, four for ethanol, and five in the remaining cases. During sucrose jelly CPP, the conditioning sessions lasted 60 min, and a piece of jelly (0.66±0.053 g) was placed in the chamber. One compartment was paired with jelly made of 10% (w/v) sucrose (Roth) in 1% (w/v) agarose (PRONA); the other, with 1% agarose. The CPP score is the difference in the amount of time spent in the drug (or sucrose jelly)-paired versus vehicle-paired chambers during the post-conditioning test. Alcohol jelly conditioned preference was conducted following a similar procedure as sucrose jelly CPP. Before the procedure, mice were offered access to both sucrose jelly and alcohol-containing sucrose jelly in addition to food and water for 3 d in order to reduce potential alcohol taste aversion. Two days after the access to jelly in the home cage ended the conditioning procedure started. One compartment was paired with jelly made with sucrose as described above and the other with jelly containing 10% (v/v) ethanol and 10% sucrose in 1% agarose. The addition of sucrose was necessary to ensure that the jelly was consumed; majority of animals did not eat unsweetened jelly.

#### T-maze

The visual cue task (VCT) and response direction task (RDT) were tested in a T-maze. During the VCT, the animals had to turn toward the arm of the maze with a visual cue (a strip of white paper, placed pseudorandomly in one of the arms). Then, mice were tested in the RDT, in which the animals had to consistently choose the same turn direction (left or right) to reach the food reward. Both tasks lasted 5 d, with two sessions per day. Each session consisted of 12 trials, and the apparatus was turned 90° clockwise every four trials to minimize the influence of extra-maze cues.

#### Social interaction

The tests were performed in a plastic cage (20.5 ×55 ×38.5 cm) that was illuminated at 50 lux. Behaviors were recorded using a camera that was placed above the cage (DMK 22AUC03, The Imaging Source). First, the experimental mouse was placed in the cage for 30 min, after which a new mouse was introduced to the cage for 10 min. After the interaction sessions were completed, the video recordings were analyzed using EthoVision XT software v11.5. For each trial, we verified that animal positions were automatically detected and manually corrected the results when necessary.

#### Saccharin preference

Mice were tested individually in cages where they had access to two 25 ml graduated drinking tubes. One tube was filled with water and the other with 0.1% saccharin solution. Food was provided *ad libitum* on the cage floor. The test lasted 24h.

#### Pavlovian approach and conditioned reinforcement

Mice were given limited access to food and kept at 85% of their initial weights at the start of the experiments. Tests were performed in operant conditioning chambers (ENV-307W, Med Associates) over 8 d. There were 25 presentations of a 10 s stimulus paired with food pellet delivery (CS+; both cue lights or 2900 Hz sound at 65 dB) and 25 presentations of a stimulus with no consequence (CS−) in pseudorandom order during each session. The assignment of sound or light as CS was counterbalanced. Then, conditioned reinforcement was tested in a single 60 min session, during which instrumental responses led to presentation of the CS+ or CS−; however, no food was delivered.

#### Operant sensation seeking

Operant responding for varied visual stimuli was conducted based on the procedure described by Olsen and Winder (2009). Conditioning chambers were equipped with photocell-equipped holes mounted 2.2 cm above the grid floor, and cue lamps (yellow light-emitting diodes) were placed 2 cm above the photocell-equipped holes (ENV-307W, Med Associates). At the beginning of each session, the house light and exhaust fan were turned on. A compound visual/auditory stimulus was presented after a mouse completed the fixed ratio [fixed response ratio 1 (FR1)], whereas exploration of the inactive holes had no consequence. The visual stimulus was a presentation of blinking lights, with a random duration of 2, 4, 6, or 8 s and a frequency of .625, 1.25, 2.5, or 5 Hz. The auditory stimulus was generated by a 65 dB tone generator (2900 Hz). The main cage light was switched off during the presentation of the stimulus. Operant sensation seeking was conducted in 1 h sessions without any prior training or dietary restriction.

#### Instrumental food self-administration

Food-restricted mice were placed in conditioning chambers for 45 min a day. Instrumental reaction (nose poke) on the active operant resulted in delivery of a food pellet (20 mg). When food self-administration reached a stable plateau (<20% change in the mean number of responses in the control group over 3 consecutive sessions), mice were subjected to progressive ratio (PR) schedule. During the 1 h PR session the number of responses required to obtain a reward increased by three each time a reward was earned (ie, 1, 3, 6, 9…). The breakpoint represents the highest number of instrumental responses performed to receive a food pellet. Then, mice were also tested under the variable interval (VI) schedule. During the 1 h VI sessions, instrumental response on the active nose poke resulted in a delivery of a food pellet. However, after each reward there was a random interval of 0–10 s in the first trial (VI10) or 0–30 s (VI30) in the second trial, during which responses on the active nose poke had no consequence. There were no cues presented during the tests.

### Statistical methods

Statistical analyses were conducted using R, GraphPad Prism, and Statistica software, significance was assessed by an ANOVA or by an appropriate two-sample test (*t* test or nonparametric). The Kolmogorov–Smirnov test was used to compare distributions of results between two samples. No data were excluded from analyses, with the exception of one rise sEPSC value, which was calculated as 0 ms. Statistics summary is given in Table 1.

## Results

### Inducible ablation of the NR1 gene in dopaminoceptive neurons

**Figure 1.**
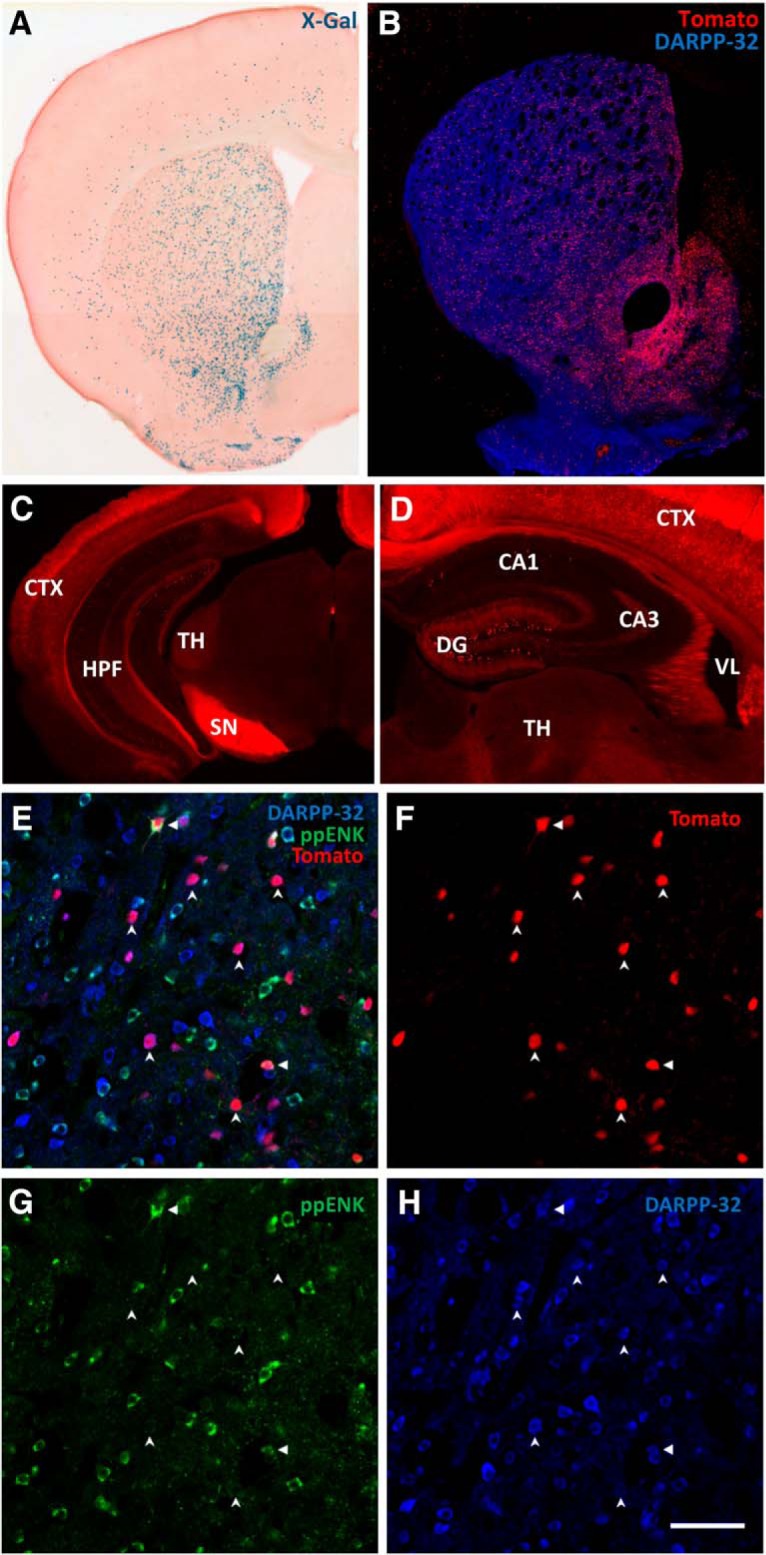
Recombinase expression in the D1CreERT2 strain. ***A***, A micrograph of a coronal section from the brain of an R26Rosa-D1CreERT2 mouse stained with X-Gal and eosin. The blue product of beta-galactosidase activity is primarily visible in the NAc and in the ventral and medial striatum. ***B***, Confocal image showing a coronal section from the brain of a tdTomato-D1CreERT2 mouse. MSNs visualized by an antibody against DARPP-32; recombined neurons, by raw tdTomato fluorescence. A strong tdTomato signal is primarily observed in the NAc and in the ventral and medial striatum. ***C***, ***D***, D1CreERT2 activity in the hippocampus and the cortex. Micrographs of coronal sections from the brain of a tamoxifen-treated tdTomato-D1CreERT2 mouse. Recombined neurons visualized by raw tdTomato fluorescence. ***E*–*H***, High-magnification of a coronal section of the NAc from a tdTomato-D1CreERT2 mouse showing tdTomato expression (***F***) in medium spiny neurons (MSNs) stained with antibodies against preproenkephalin (***G***; ppEnk) and DARPP-32 (***H***) and their composite image (***E***). Vertical arrows indicate examples of DARPP-32+, Proenkephalin−, and tdTomato+ cells (ie, recombined direct-pathway MSNs), whereas horizontal arrows indicate triple-positive cells (ie, recombined indirect pathway MSNs). Scale bar, 50 µm. CA1, Field CA1; CA3, field CA3: CPu, caudoputamen; CTX, cerebral cortex; DG, dentate gyrus; HPF, hippocampal formation; SN, substantia nigra; TH, thalamus; VL, lateral ventricle.

The cell-type-specificity of D1CreERT2 recombinase (Rodriguez Parkitna et al., 2010) expression was assessed by crossing with the ROSA26-lacZ (Soriano, 1999) and ROSA26-tdTomato (Madisen et al., 2010) reporter strains. Tamoxifen-induced recombination was primarily observed in the NAc and in the medial striatum. A lower density of recombined cells was also visible in the dorsal and lateral striatum and in discrete areas of the cortex and the ventral hippocampus (Fig. 1*A*–*D*). The observed pattern of recombination matches the distribution of D1 receptor expression in the rodent brain, though the extent of recombination in the dorsal striatum was lower than could be anticipated (Fremeau et al., 1991). In the NAc, recombination occurred in DARPP32-positive and predominantly proenkephalin-negative cells, thus, primarily in the medium spiny neurons of the direct pathway (Fig. 1*E*–*H*). Although the cell-type-specificity was the same as observed in other transgenic mice using the same D1 promoter (Rodriguez Parkitna et al., 2010; Rodriguez Parkitna et al., 2013; Bilbao et al., 2014), we note that the activity of the transgene in the dorsal striatum appears to be strain-dependent. It was consistent with the pattern previously reported in the *Srf^D1CreERT2^* line (Rodriguez Parkitna et al., 2010), but less extensive compared with the mGluR5^D1-KD^ line (Rodriguez Parkitna et al., 2013).

**Figure 2.**
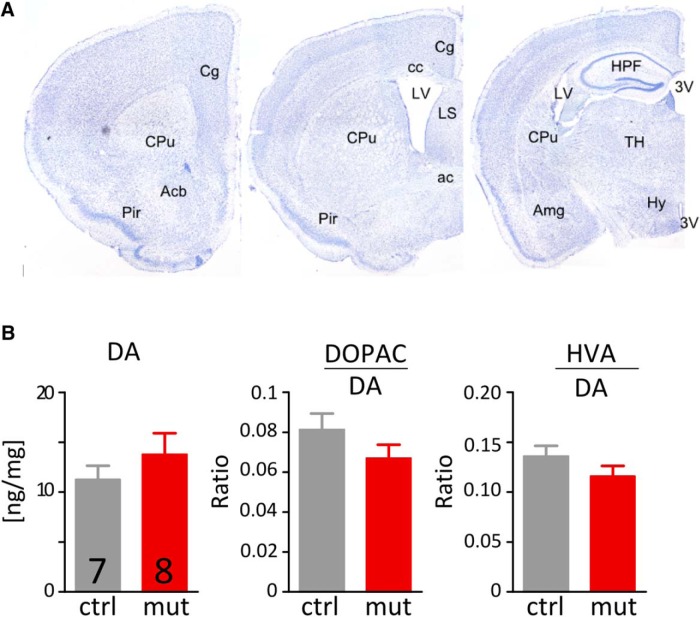
Characterization of the NR^D1CreERT2^ line. ***A***, Normal anatomical features in coronal sections from brains of mutant mice stained with cresyl violet. ***B***, Dopamine content and turnover in the striatum. The striatum (including NAc) was dissected and assayed for the levels of dopamine and it’s metabolites. Each bar represents a mean from five samples, error bars show SEM values. There were no significant differences between sample means (*t* test). 3V, Third ventricle; ac, anterior commissure; Acb, nucleus accumbens; Amg, amygdala: cc, corpus callosum; Cg, cingulate gyrus; CPu, caudoputamen; HPF, hippocampal formation; Hy, hypothalamus; LV, lateral ventricle; LS, lateral septum; Pir, piriform area; TH, thalamus.

**Figure 3.**
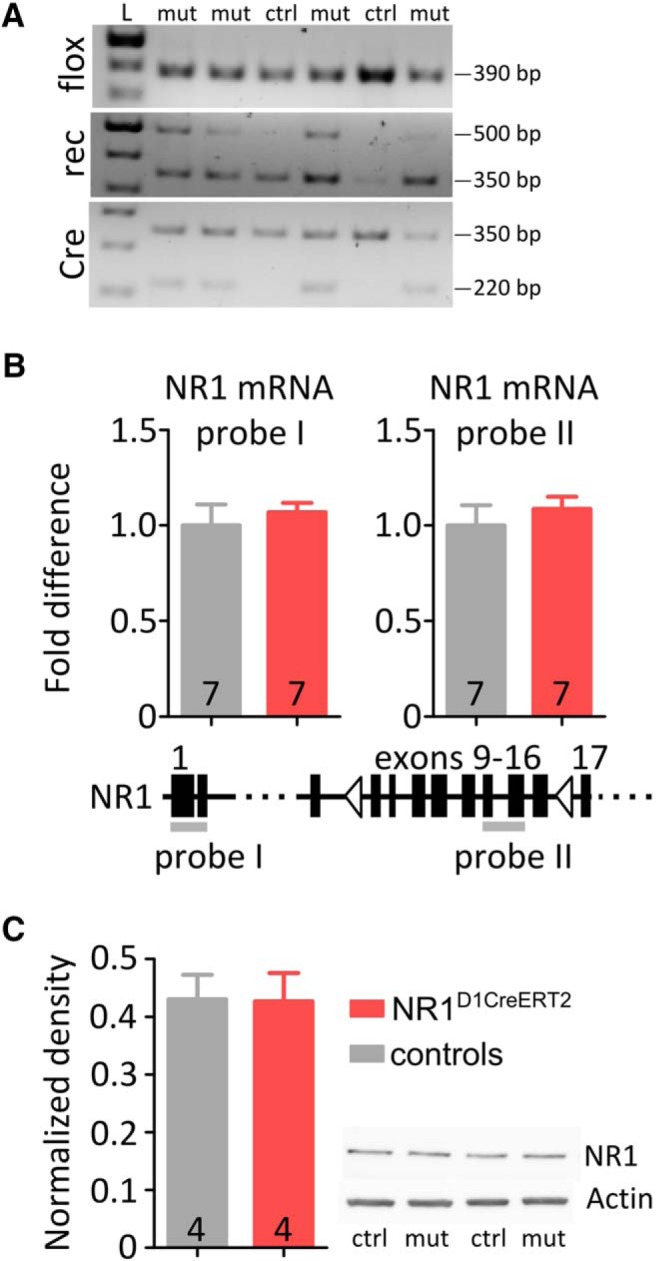
NR1 recombination and expression. ***A***, PCR detection of the recombined NR1 allele in genomic DNA samples from the NAc and striatum of NR1^D1CreERT2^ mice and controls. The “flox” panel shows the presence of a single 390 bp band, confirming the flox/flox genotype. In the “rec” panel, the presence of a ∼500 bp band confirms recombination between the loxP sites, whereas the 350 bp band is an internal positive control. In the “Cre” panel, the 220 bp band confirms the presence of the D1CreERT2, and the 350 bp band is a positive control. ***B***, Abundance of NR1 mRNA, as assayed by qPCR. The bar graphs show the relative abundance measured using probes targeting a non-recombined gene fragment (probe I) and the potentially deleted fragment (probe II; see the diagram below the graphs). The numbering of exons is based on the gene sequence ENSMUSG00000026959 and on the transcript ENSMUST00000028335. ***C***, NMDA receptor subunit protein abundance detected by Western blotting and normalized to actin. A representative result is shown on the right. Error bars show SEM values, sample sizes are indicated on the bar graphs. There were no significant differences between sample means (t-test).

The NR1^D1CreERT2^ strain was generated by crossing D1CreERT2 mice with a strain carrying a floxed variant of the NR1 (*Grin1*) gene (Niewoehner et al., 2007). The CreERT2 normally remains in the cytosol, until treatment with tamoxifen, which enables translocation of the recombinase to the nucleus and recombination of the target sequence. One month after the recombinase was induced to cause deletion in the NR1 gene of the exons encoding the transmembrane region of the protein, the general anatomy of the primary areas expressing D1 receptors appeared unaltered (Fig. 2*A*) and the dopamine levels and turnover were not affected (Fig. 2*B*). The presence of the recombined NR1 gene in the striatum/NAc was confirmed by PCR using genomic DNA with primers flanking the entire floxed region of the NR1 gene (Fig. 3*A*). Despite the deletion, the total abundance of the NR1 transcript and protein in the homogenized striatum were similar in control and mutant mice (Fig. 3*B*,*C*). Based on the apparent protein mass in the Western blot and abundance of mRNA corresponding to the recombined NR1 gene fragment, the observed levels corresponded exclusively to the non-recombined form (Table 1).

### Loss of functional NMDA receptors and its effect on LTP

**Figure 4.**
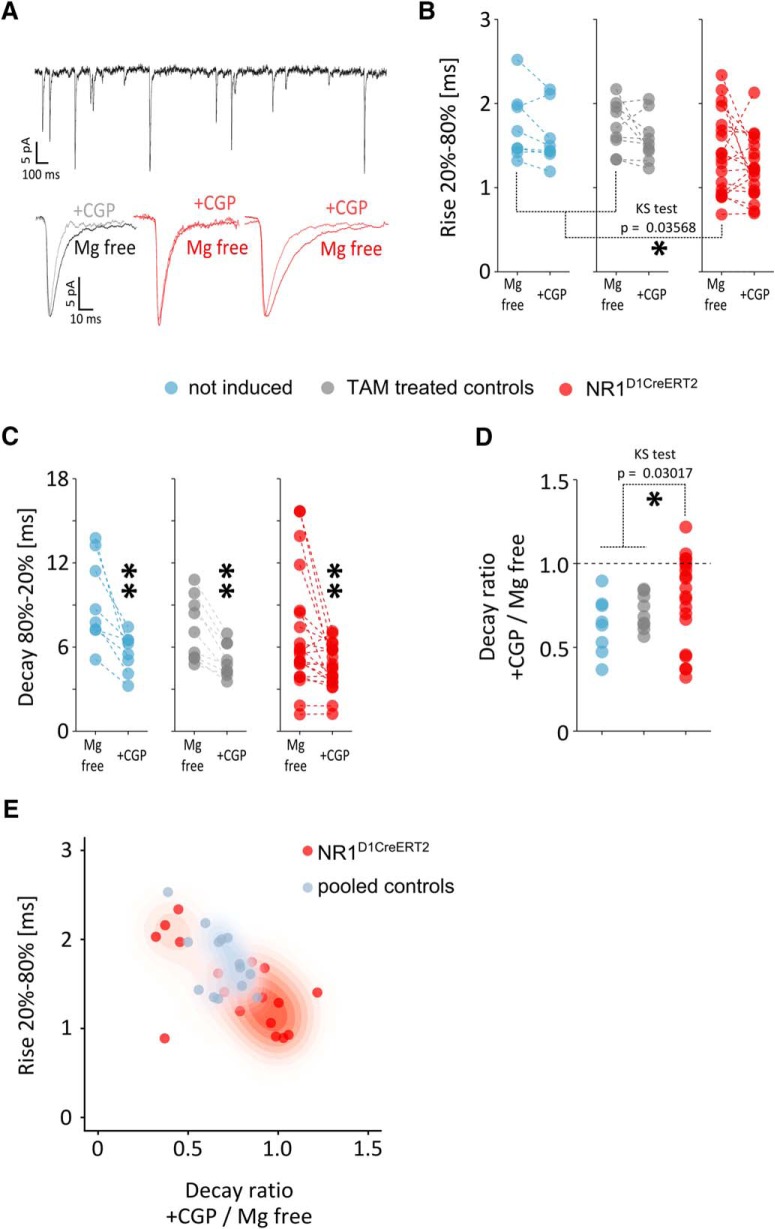
Whole-cell patch-clamp analysis of NAc cells. ***A***, Representative sEPSC traces and averaged sEPSCs before and after application of 10 µm CGP37849: a control (grey, left), a mutant lacking NMDA antagonist sensitivity (red, middle), and a mutant with NMDA antagonist sensitivity (red, right). ***B***, Comparison of mean times elapsed between reaching 20% and 80% of the maximum amplitude (“rise”) before and after CGP37849 application. Dashed lines connect the data points representing the same neuron. The “not induced” mice were animals with the NR1^D1CreERT2^ genotype that were not treated with tamoxifen. A significant difference was observed by the Kolmogorov–Smirnov test in the current rise times between neurons from NR1^D1CreERT2^ mice and both controls combined; *p < 0.05. ***C***, Comparison of mean times elapsed when decreasing from 80% to 20% of the maximum amplitude (“decay”), before and after CGP37849 application. Statistical significance with the paired Wilcoxon test at **p < 0.01; V = 36, *p* = 0.0078, V = 45, *p* = 0.0039 and V = 184, *p* = 0.0020, respectively. ***D***, Analysis of the NMDA antagonist effect. The “decay ratio” was calculated as the value after perfusion with CGP37849 divided by the initial decay in Mg^2+^-free conditions. A significant difference was observed by the Kolmogorov–Smirnov test in the distribution of results between neurons from NR1^D1CreERT2^ mice and both controls combined; *p < 0.05. ***E***, Relation between the rise and the decay ratio. Control groups are pooled and shown as blue-grey, data from NR1^D1CreERT2^ mice is shown in red. The shading represents approximate distribution of density calculated with the kde2d function from the MASS R package, with darker shades corresponding to respective higher relative densities.

To assess the NMDA receptor activity, we performed whole-cell recordings of sEPSCs from neurons in the NAc core of tamoxifen-treated NR1^D1CreERT2^, control, and non-induced NR1^D1CreERT2^ mice (Fig. 4*A*). Treatment with CGP37849, which is an NMDA receptor antagonist, had no significant effect on the rise time but caused a reduction in the current decay time (Fig. 4*B*,*C*). Nine of the 21 neurons assayed in the mutant animals were insensitive to the NMDA antagonist and exhibited a reduction in decay time of <10%, whereas for all controls (17 neurons total), the reduction was 10% or greater. The lack of the NMDA receptor component of the current confirms the presence of the mutation. Some of the neurons in mutant mice showed an opposite trend, with >50% reduction in the averaged sEPSC decay time after antagonist treatment in five cases. Therefore, no difference was observed between the mean level of the sEPSC decay time after antagonist treatment in mutant versus control animals; however, a significant difference in the distribution of results was observed (Fig. 4*D*). The peak rise times in NAc neurons in tamoxifen-treated NR1^D1CreERT2^ mice were significantly shorter than the peak rise times in NAc neurons in controls (Fig. 4*B*). The short rise times correlated with the lack of NMDA antagonist sensitivity (Fig. 4*E*).

**Figure 5.**
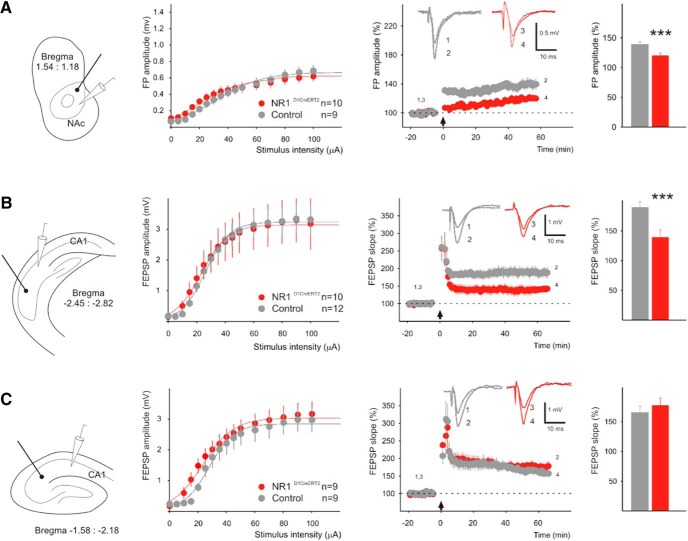
LTP in the NAc (*A*), the ventral hippocampus (*B*), and the dorsal hippocampus (*C*). The diagrams on the left show the placement of the stimulating electrode (thick line with a dot) and the recording pipettes (white cone). The graphs in the middle show the relation between the stimulus intensity and fEPSP amplitude. The panels on the right show the relative amplitude (***A***) or slope (***B***, ***C***) values of the FPs. The use of amplitude for nucleus accumbens (NAc) was necessitated by the relatively small potential amplitudes and the resulting low signal-to-noise ratio. The black arrow indicates the time point at which HFS was applied. Insets, Representative evoked potentials in control and NR1^D1CreERT2^ mice before and after stimulation, as indicated by the numbers 1–4. The bar graphs on the right show mean averaged values 45–60 min after HFS. Significant differences (*t* test) are indicated with ***p < 0.001, and error bars represent the SEM. The *t* test values are: *t* = 21.719, *p* < 0.0001; t = 66.892, *p* < 0.0001; *t* = −0.729, *p* = 0.473, respectively. Sample sizes are indicated in the legends.

To assess the effects of the mutation on synaptic plasticity, we performed LTP measurements in the core of the NAc and in the two regions of the hippocampus (Fig. 5). The mutation had no effect on the relation between the stimulation intensity and the FP magnitude, indicating a lack of modifications in the basal synaptic transmission. However, compared with control preparations, the LTP was attenuated in the NAc of the NR1^D1CreERT2^ mice (138 ± 6 vs 118 ± 5% of the baseline between 45 and 60 min after HFS, respectively; Fig. 5*A*). A reduction in the LTP magnitude was also observed in the ventral hippocampus, in the area where recombination was observed in NR1^D1CreERT2^ mice (Fig. 5*B*; 189 ± 14 vs 140 ± 12%). In contrast, in the dorsal hippocampus, which has little D1 expression and no observed Cre recombinase activity, the LTP magnitudes in slices obtained from the mutant and the control mice were similar (Fig. 5*C*; 165 ± 11 vs 177 ± 13%).

### Reward-conditioned behaviors in NR1^D1CreERT2^ mice

**Figure 6.**
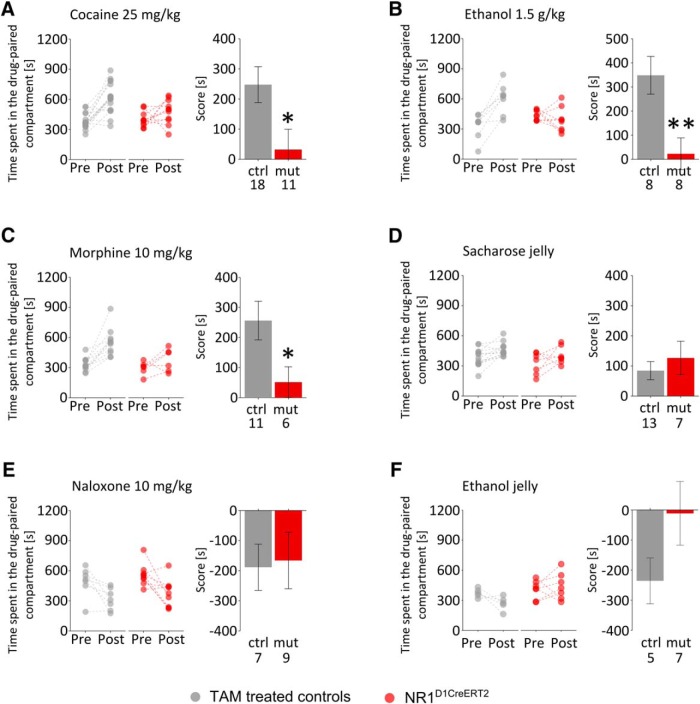
CPP. ***A***, Drug CPP with cocaine (25 mg/kg), (***B***) ethanol (1.5 g/kg), and (***C***) morphine (10 mg/kg). Panels on the left show the time spent in drug-paired compartments during pre- and post-conditioning tests. Dashed lines connect points representing the same animal. Panels on the right show the preference score, which is the difference in time spent in the two compartments during the post-conditioning test. Score difference comparison: *t* = 2.40, *p* = 0.0250; *t* = 2.47, *p* = 0.0263; and *t* = 3.19, *p* = 0.0068, respectively. ***D***, Sucrose jelly CPP, score difference comparison: *t* = −0.66, *p* = 0.5237. ***E***, Naloxone-conditioned (10 mg/kg) place aversion, score difference comparison: *t* = −0.18, *p* = 0.8563. ***F***, Sucrose jelly with alcohol versus sucrose jelly preference, score difference comparison: *t* = 1.582, *p* = 0.1447. Sample sizes are indicated on the bar graphs in each panel, and error bars show SEM values. Significant differences (*t* test) are indicated with *p < 0.05 and **p < 0.01.

We had anticipated that the selective loss of NMDA receptors on D1-expressing neurons should prevent drug-induced plasticity of direct-pathway NAc neurons and, therefore, reduce the ability to acquire conditioned behaviors. We tested CPP, which depends on the ability to form an association between a context and a reward. Control animals readily acquired CPP for a context associated with intraperitoneal injections of 25 mg/kg cocaine, whereas the NR1^D1CreERT2^ mice failed to acquire a preference (Fig. 6*A*; repeated-measures two-way ANOVA; genotype: *F*_(1,27)_ = 4.05, *p* = 0.0542; test: *F*_(1,27)_ = 34.235, *p* < 0.0001; genotype × test: *F*_(1,27)_ = 6.537, *p* = 0.0165). A similar result was observed for other addictive drugs. Mutant mice showed no preference to a compartment paired with 1.5 g/kg alcohol (Fig. 6*B*; genotype: *F*_(1,14)_ = 1.51, *p* = 0.239; test: *F*_(1,14)_ =10.10, *p* = 0.0067; genotype × test: *F*_(1,14)_ = 19.58, *p* = 0.0006) or with 10 mg/kg morphine (Fig. 6*C*; genotype: *F*_(1,15)_ = 7.704, *p* = 0.0141; test: *F*_(1,15)_=27.045, *p* = 0.0001; genotype × test: *F*_(1,15)_ = 4.248, *p* = 0.0571), although both drugs induced a robust preference in the littermate controls.

Strikingly, this deficit was specific to the effects of drugs of abuse. Mutant mice acquired a preference for a compartment where they could consume sucrose jelly (Fig. 6*D*; genotype: *F*_(1,18)_ = 3.702, *p* = 0.0703; test: *F*_(1,18)_ = 10.646, *p* = 0.0043; genotype × test: *F*_(1,18)_ = 0.001, *p* = 0.9775) and acquired normal conditioned place aversion to a compartment paired with intraperitoneal injections of 10 mg/kg naloxone, which is an opioid antagonist (Fig. 6*E*; genotype: *F*_(1,14)_ = 1.759, *p* = 0.206; test: *F*_(1,14)_ = 17.63, *p* = 0.0009; genotype × test: *F*_(1,14)_ = 0.01, *p* = 0.923). Finally, there was a significant effect of the mutation on preference between sucrose jelly and alcohol-containing sucrose jelly, without significant pretest–post-test difference in preference (Fig. 6*F*; genotype: *F*_(1,10)_ = 6418, *p* = 0.0297; test: *F*_(1,14)_= 0.7934, *p* = 0.3940; genotype × test: *F*_(1,14)_ = 3.160, *p* = 0.1058). The observed difference in score during the post-test was also not significant, though the trend suggested lack of conditioning in NR1^D1CreERT2^ mice.

**Figure 7.**
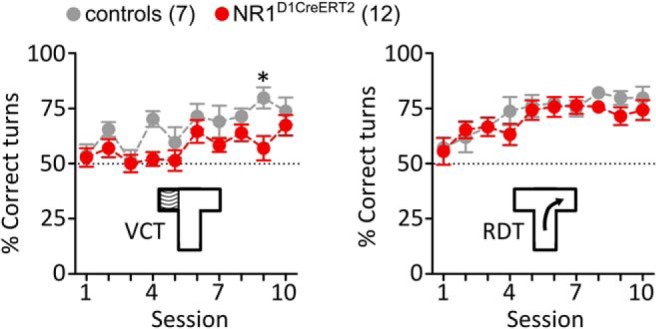
VCT and RDT task in the T-maze. The graphs show the mean fraction of turns toward the rewarded arm per session (12 trials). The diagram inserts summarize the strategy required to reach the food pellet in the two phases. Group sizes are indicated in the legend, and error bars show SEM values. Significant differences (Bonferroni corrected *t* test) are indicated with *p < 0.05.

Inputs from the hippocampus to the NAc were implicated in the spatial learning and flexibility required for goal-directed behavior (Goto and Grace, 2005), which could partly explain the CPP phenotype in NR1^D1CreERT2^ mice. To test this possibility, we used a T-maze task, which requires an allocentric or egocentric strategy to reach a food pellet. We found that the NR1^D1CreERT2^ mice were slower to improve their performance in the visual-cue task (VCT; Fig. 7; session: *F*_(9,153)_ = 4.031, *p* = 0.0001; genotype: *F*_(1,153)_ = 18.07, *p* = 0.0005; session × genotype: *F* = 0.9686, *p* = 0.4682). After the shift to the response direction task (RDT), animals of both genotypes performed equally.

**Figure 8.**
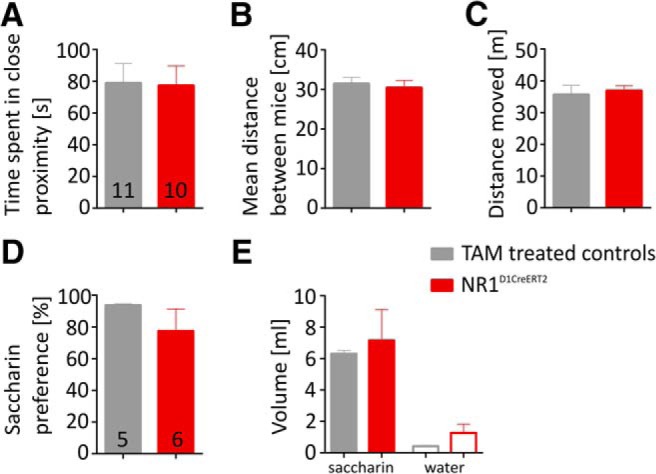
Social interaction and saccharin preference tests. ***A***, The graph shows the time spent in close proximity between interaction partners, (***B***) the mean distance between animals during test, and (***C***) total distance moved by mice during the social interaction test. ***D***, Saccharin preference after and (***E***) the volume of saccharin and water that animals drank during 24 h. Group sizes are indicated in the bar graphs, and error bars show SEM values.

We also tested social interaction and saccharin preference, behaviors that involve sensitivity to natural rewards but do not involve conditioning. Although social interaction is often used to test anxiety levels (File and Seth, 2003), the contact between animals also has a reward component (Trezza et al., 2011) and is dependent on dopamine signaling (Gunaydin et al., 2014; Matthews et al., 2016). We found that NR1^D1CreERT2^ mice spent the same amount of time as controls in close contact with an unknown conspecific and maintained the same average distance with the other mouse (Fig. 8*A*,*B*). Mice did not differ in the total distance moved during the experiment (Fig. 8*C*). Likewise, in the saccharin preference test, which was also shown to depend on the activity of the dopamine system (Tye et al., 2013), mutant mice showed the same preference for drinking the saccharin solution (Fig. 8*D*,*E*). Together these results show no effect of the mutation in tests involving a social or gustatory reward, and also no indication of increased anxiety or anhedonia.

### Appetitive pavlovian and instrumental conditioning

**Figure 9.**
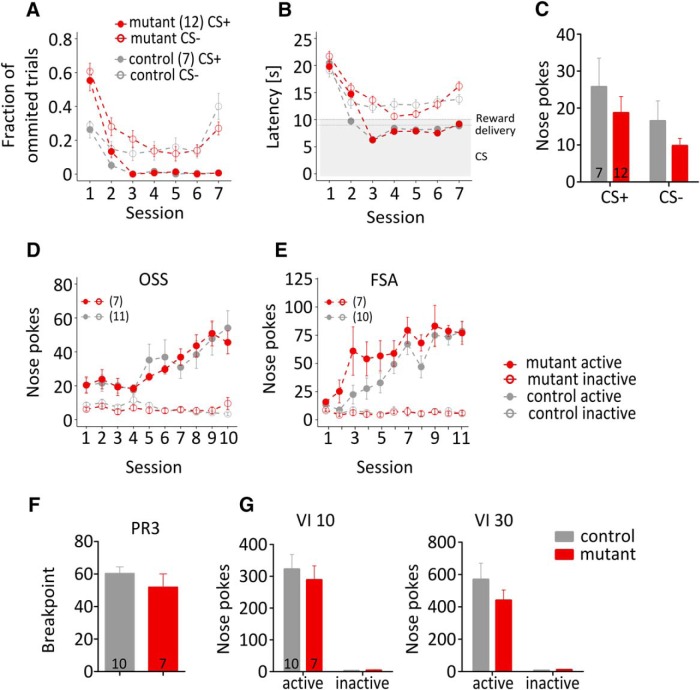
Pavlovian and instrumental conditioning. ***A***, The graph shows the fraction of ignored cue presentations (no approach to the food dispenser before the next cue). ***B***, Latency to approach the food dispenser (excluding omitted trials). The grey area corresponds to cue presentation. The dashed lines show activation of the food dispenser. ***C***, Conditioned reinforcement. Numbers of instrumental responses (nose pokes) linked to presentation of the CS+ or CS−. ***D***, OSS. ***E***, Instrumental food self-administration (FSA). ***F***, PR schedule of reinforcement. The number of responses required to obtain a food pellet increased by three after each reward. The breakpoint is the highest number of responses an animal performed to obtain a pellet. ***G***, VI schedule. Group sizes are indicated in the legends or in the panels, as appropriate. Error bars show SEM values.

We expected that the loss of NMDA receptors in the NAc and parts of the striatum could affect the ability to learn contingencies between stimuli or actions and their outcomes. However, both the control and NR1^D1CreERT2^ mice acquired the pavlovian-approach behavior triggered by presenting a conditioned stimulus (CS+) predicting the delivery of a food pellet. After only three sessions, the mice would approach the food magazine during every trial when the CS+ was presented, and the mean latency of the head entry into the magazine was timed immediately before the food dispenser was activated (Fig. 9*A*,*B*). Then, animals were tested for instrumental responding for the CS+ and CS− to assess whether the stimulus became a conditioned reinforcer. Animals performed significantly more responses for the CS+, without a significant effect on the genotype (Fig. 9*C*; CS: *F*_(1,19)_ = 6.153, *p* = 0.0227; genotype: *F*_(1,19)_ = 1.452, *p* = 0.243; genotype × CS: *F*_(1,19)_ = 0.002, *p* = 0.9657).

A second cohort of naïve mice was tested in an operant sensation-seeking test (OSS). In this test, the active nose poke triggers a random sequence of blinking lights and speaker sounds. Both NR1^D1CreERT2^ mice and controls performed operant responses that led to the presentation of sensory stimuli, whereas only a few nose pokes were performed on the inactive hole (Fig. 9*D*; three-way repeated-measures ANOVA; main factors: session: *F*_(9,144)_ = 5.976, *p* < 0.0001; active/inactive: *F*_(1,16)_ = 58.986, *p* < 0.0001; genotype: *F*_(1,16)_ = 0.047, *p* = 0.831). Hence, the mutation did not diminish a sensation-seeking-like trait or sensitivity to sensory stimuli.

A third cohort of mice was tested for instrumental food self-administration. Under a FR1 schedule, both the control and NR1^D1CreERT2^ mice acquired instrumental responding for food, reaching 78.5±9.1 and 69.1±11.6 responses per session, respectively (Fig. 9*E*). The motivation to obtain food under a progressive ratio schedule (PR3) was not affected by the mutation (Fig. 9*F*; breakpoints 60.3±4.1 and 51.9±8.2 for control and NR1^D1CreERT2^, respectively). Additionally, responding under a VI schedule with a random interval (0–10 or 0–30 s) after each reward was normal in NR1^D1CreERT2^ mice (Fig. 9*G*).

## Discussion

We find that NR1^D1CreERT2^ mice lack the ability to associate contextual cues with the rewarding effects of drugs of abuse, with minor or no deficits in other reward-conditioned behaviors or learning in general. Based on the results from reporter strains, the mutation was observed in areas where D1 receptors are expressed and had good cell-type-specificity (ie, ppEnk-, DARPP-32+ neurons). High efficiency of the recombinase was observed in the NAc, and the density of targeted neurons in the dorsolateral striatum was visibly lower. In the NAc core of NR1^D1CreERT2^ mice, the proportion of neurons lacking the NMDA-dependent component of sEPSCs was ∼40%. Consistent with expectations, the loss of functional NMDA receptors diminished the LTP magnitude, assessed with the use of the field potential recording method, in the NAc core (Schotanus and Chergui, 2008) and in the ventral, but not dorsal, hippocampus, which is also consistent with the pattern of recombinase activity and D1 expression. The decreased LTP is also consistent with reported effects of striatum-specific NR1 inactivation (Dang et al., 2006). Curiously, we observed no decrease in NR1 protein or transcript abundance in the striatum/NAc. Because the presence of recombined NR1 alleles in the striatum was confirmed on genomic level and the electrophysiological data is consistent with loss of NMDA receptors in part of striatal cells we assume that expression of NR1 is likely increased in those striatal neurons, which retained intact NR1. These could be D2-expressing neurons lacking Cre expression or possibly D1-expressing neurons where recombination did not occur. The possibility that the observed protein and mRNA levels are due to the presence of a truncated product of the NR1 gene should be excluded based on the protein size and presence of the floxed fragment in the NR1 transcript. This observation, together with the presence of a subpopulation of neurons expressing a stronger than usual NMDA receptor-dependent component in the sEPSCs in the NAc, may indicate a compensatory increase in NMDA receptor abundance occurring in non-targeted neurons. An increase in receptor abundance would not be a developmental adaptation, because the mutation was induced after mice had reached 8–10 weeks of age. Also, it would be compensatory in the meaning that it could restore the total protein levels in the striatum but not necessarily restore normal function in the reward system. An increase in NMDA receptor abundance in D2-expressing medium spiny neurons could actually be a contributing factor in some of the observed phenotypes. In summary, these findings may support the existence of a mechanism balancing NMDA receptor activity among D1-expressing neurons or between the D1- and D2-expressing neurons, which was previously proposed based on the effects of NR1 deletion on amphetamine sensitization (Beutler et al., 2011b).

Based on previous studies, we anticipated that the selective loss of NMDA receptors on D1 neurons should broadly affect associative learning (Yin et al., 2008) and impair instrumental responding (Beutler et al., 2011a), especially under complex schedules (Jin and Costa, 2010). However, the results were inconsistent with this hypothesis. Mutant mice acquired the Pavlovian approach behavior normally and showed conditioned reinforcement to the CS+. Furthermore, instrumental conditioning with natural rewards was also not affected. NR1^D1CreERT2^ mice showed normal responses for a stimulus previously associated with a food reward and performed similarly to controls in both the OSS and instrumental food self-administration under all reinforcement schedules tested. Moreover, the mutation had no effect on social interaction or sweet taste preference, both of which depend on reward system activity. The only observed deficit was in the VCT, where the moderately inferior performance is consistent with the effects of inducible inactivation of D1-expressing neurons by tetanus-toxin expression in mice (Yawata et al., 2012) and is partially consistent with the reported effects of pharmacological inactivation of hippocampal inputs on D1-expressing neurons in the NAc in rats (Goto and Grace, 2005). Unlike the latter report, we observed no change in the RDT.

The NR1^D1CreERT2^ mice do not acquire drug-induced CPP. This phenotype is partly similar to the effects of striatal infusion of an NMDA antagonist (Cervo and Samanin, 1995; Popik and Kolasiewicz, 1999) and the reduced cocaine CPP reported in animals expressing a calcium-impermeable NR1 variant in D1-expressing neurons (Heusner and Palmiter, 2005). In our case, even a high (25 mg/kg) dose of cocaine completely failed to induce a preference. Furthermore, we show that the phenotype is not limited to the effects of psychostimulants. NR1^D1CreERT2^ mice did not develop preference for a compartment paired with ethanol or morphine injections. However, the phenotype does not represent a general impairment of associative learning, because sucrose-induced CPP or naloxone-induced CPA in the same experimental cages was normal. Additionally, although animals showed no preference for the context associated with alcohol jelly, there was a genotype effect on the change in time spent in the alcohol jelly compartment between the pretest and post-test. It should be noted though that in this experiment agarose was additionally sweetened with sucrose to ensure that animals ate the jelly.

The selectivity of the effect of the mutation on CPP is striking and suggests that NMDA receptor activity in D1-expressing neurons is essential for associating the drug effects with their context but possibly redundant for a natural reward or for an aversive stimulus. This selectivity may result from the presence of NMDA receptors in D1-expressing neurons in some areas (eg, the dorsolateral striatum), which may be sufficient for non-drug rewards. This result implies that parallel and partly independent systems underlie positive reinforcement and is consistent with the reported influence of NMDA-dependent plasticity in D1-expressing neurons in the NAc shell on the response to cocaine-associated, but not food-associated, cues (Pascoli et al., 2014). Another possibility is that the lack of drug-induced CPP is actually associated with altered activity of neurons outside the basal ganglia. The major inputs to the NAc, the amygdala, prefrontal cortex, and hippocampus all receive dopamine inputs and contain D1-expressing neurons. Altered plasticity caused by loss of NMDA receptors (as for instance impaired LTP in the ventral hippocampus) could affect learning during the CPP procedure. However, because previous reports concur that injection of an NMDA antagonist into the NAc was sufficient to prevent drug-induced CPP (Cervo and Samanin, 1995; Popik and Kolasiewicz, 1999) we find this possibility less likely.

In summary, we find that NMDA receptor-dependent signaling on dopaminoceptive neurons expressing D1 receptors is necessary for the association of drugs of abuse with the context of their effects. However, the selective loss of NMDA receptors had no appreciable effect on the memory of the context of a natural reward or an aversive stimulus, and did not cause a change in sensitivity to natural rewards.

**Table 1.** Statistics summary

	Data structure	Type of test	95% confidence intervals
Figure 2B, DA	assumed normal distribution	two-sample t test (equal variance)	(−7.812, 2.823)
Figure 2B, DOPAC/DA	assumed normal distribution	two-sample t test (equal variance)	(−0.008933, 0.03776)
Figure 2B, HVA/DA	assumed normal distribution	two-sample t test (equal variance)	(−0.01168, 0.05225
Figure 3B, left graph	assumed normal distribution	two-sample t test (equal variance)	(−0.3303, 0.1928)
Figure 3B, right graph	assumed normal distribution	two-sample t test (equal variance)	(−0.3542, 0.1798)
Figure 3C	assumed normal distribution	two-sample t test (equal variance)	(−0.1550, 0.1628)
Figure 4B	no assumptions	two-sample Kolmogorov–Smirnov	no confidence intervals
Figure 4C, not-induced	assumed symmetry around the median	Wilcoxon signed rank test	(1.4670, 6.3505)
Figure 4C, TAM treated	assumed symmetry around the median	Wilcoxon signed rank test	(1.038, 3.582)
Figure 4C, NR1^D1CreERT2^	assumed symmetry around the median	Wilcoxon signed rank test	(0.384, 4.348)
Figure 4D	no assumptions	two-sample Kolmogorov–Smirnov	no confidence intervals
Figure 5A	assumed normal distribution	Two-sample *t* test (equal variance)	(17.924, 21.602)
Figure 5B	assumed normal distribution	Two-sample *t* test (equal variance)	(47.978, 51.000)
Figure 5C	assumed normal distribution	Two-sample *t* test (equal variance)	(−21.381, 44.754)
Figure 6A, cocaine score comparison	assumed normal distribution	Welch *t* test	(29.66433, 401.93406)
Figure 6B, morphine score comparison	assumed normal distribution	Welch *t* test	(28.77745, 396.71346)
Figure 6C, morphine score comparison	assumed normal distribution	Welch *t* test	(105.9197, 545.6453)
Figure 6D, sucrose jelly score comparison	assumed normal distribution	Welch *t* test	(−184.3338, 100.2870)
Figure 6E, naloxone score comparison	assumed normal distribution	Welch *t* test	(−282.3449, 237.6449)
Figure 6F, alcohol jelly score comparison	assumed normal distribution	Welch *t* test	(−540.5, 91.60)
Figure 8A	assumed normal distribution	two-sample *t* test (equal variance)	(−37.92, 34.74)
Figure 8B	assumed normal distribution	two-sample *t* test (equal variance)	(−6.048, 3.976)
Figure 8C	assumed normal distribution	two-sample *t* test (equal variance)	(−8.227, 5.784)
Figure 8D	assumed normal distribution	two-sample *t* test (equal variance)	(−18.20, 50.83)

## Synthesis

The decision was a result of the Reviewing Editor Karen Szumlinski and the peer reviewers coming together and discussing their recommendations until a consensus was reached. A fact-based synthesis statement explaining their decision and outlining what is needed to prepare a revision is listed below. The following reviewers agreed to reveal their identity: Devin Mueller, L. Chandler

Synthesis:

The report eN-NWR-0084-15, entitled "NMDA receptors in dopaminoceptive neurons are crucial for drug-induced conditioned place preference but not for appetitive learning in general" was deemed potentially acceptable for publication in eNeuro pending revisions as outlined below. This decision was reached following consultation with 2 reviewers, one with expertise in behavioral pharmacology and place-conditioning and the other with expertise in the neuropharmacology and neurophysiology of NMDA receptors. Both reviewers agreed that the study was of reasonably high significance but both had issue with data presentation, including the outright lack of statistical results and the lack of clarity in the graphical and textual presentation of findings. I also concur with Reviewer 1 that the hypothesis should be more clearly stated and developed in the Introduction and agree with Reviewer 2 that a discussion regarding potential developmental compensation of other dopamine receptors should be included. I also tend to agree with Reviewer 2 with respect to the distinctions between the place-conditioning procedures for drugs of abuse vs. food and suggest that the authors either consider assaying the mutant mice in another paradigm (e.g., conducting a simple alcohol drinking study) to determine whether or not the GluN1 mutants are devoid of drug reward or have perturbations in drug-related associative learning. Having consulted with both reviewers on this report, I am of the opinion that this report would be improved by the inclusion of additional experimentation to directly address this remaining issue. Related to this, Reviewer 1 and I both agree that the title of the article should be revised as only 1 alternative test for appetitive conditioning was conducted (and "in general" implies that a battery of tests were run) and there is no mention of the conditioned aversion study. Thus, we are all in agreement that major revisions are required on this report in order to render it more acceptable for publication in eNeuro.

Reviewer 1:

In the present study, the authors examined the necessity of NMDA receptors in D1 receptor-expressing neurons in appetitive and drug-induced learning. Using transgenic mice in which inducible knockdown of the NR1 subunit of NMDA receptors in D1-expressing neurons was achieved, the authors found that NMDA receptors in D1-expressing neurons mediated cocaine-, morphine-, and alcohol-induced conditioned place preference. NR1 knockdown, however, did not affect preference for a food-associated chamber or instrumental food seeking, nor did it affect naloxone-induced conditioned place aversion. These results suggest that NMDA receptors in D1-expressing neurons mediate the rewarding effects of drugs of abuse, but not natural rewards.

These findings are interesting as they demonstrate that a subset of neurons containing both D1 and NMDA receptors are responsible for drug-induced learning. However, I have some concerns that must be addressed.

1. In the introduction, there is no clear hypothesis or rationale for the study. Instead the authors simply highlight the use of their transgenic mouse without indicating what they expect to contribute.

2. There are no statistics provided in the results, figure captions, or statistical chart for the data presented in Figures 2B, 3B and 3C.

3. Minor point: There is a repeated sentence on p.8 line 234.

Reviewer 2:

This is a reasonably interesting manuscript which involves generation of a transgenic mouse with inducible inactivation of the NR1 receptor selectively on D1 expressing neurons. As would be expected, electrophysiological assessment of NMDA receptor sensitive neurons in the NAc of the transgenic mouse revealed approximately half of the neurons lacked NMDA receptor sensitivity. Presumably the remaining 50% reflected mostly D2 expressing neurons. Upon assessment of the phenotype , it was observed that inducible knockout of NR1 on D1 expressing neurons resulted in context specific drug memory using CPP without altering the reward memory for food and did not alter the aversive memory of naloxone. In general, the experimental results appear solid, but there are several concerns that need to be addressed.

1) For the CPP presented in Figure 6, please clarify whether there was a main effect of time (pre vs post). This is especially important for the data in Figure 6B.

2) Since the main finding of the paper is the selective loss of CPP for addictive drugs but not food, the assessment of this would benefit from additional evidence. There is some concern that the CPP for drugs vs food is a bit of comparing apples to oranges (e.g. for drug induced CPP the drugs are administered prior to being place in the box whereas the food preference involves the consumption of food during testing (and thus introduces an appetitive component). It would be more convincing if similar appetitive aspects were included in the assessment of drug CPP, or some complete different assessment of drug vs food reward was examined.

3) The data presented in Figure 6A,B and C is very difficult to see. I think would be much better is there data for the controls and mutants were broken out as separate graphs similar to how the data was presented in Figure 4. There is a similar concern with the line graphs in Figure 8. In particular the open graph circles. Perhaps the lines of the circles could be make darker/thicker.

4) In the description of the results of the VCT data presented in figure 7, it is stated that the mutant mice exhibited slower learning, but there were similar levels of reward choice by the 10th session. I agree that the there is a main effect of genotype, but the data does not appear to show that the effect had normalized by session 10. In fact, it almost looks cyclical with the largest difference observed at 4 and 9. This is a relatively minor point and it would be best to just leave it as a genetypic difference.

5) The fact that the level of NR1 expression did not change is somewhat odd. The authors discuss reasonable potential explanations for this, but perhaps this could benefit from a bit more discussion of how D2 neurons could compensate for the loss of NR receptors on D1 neurons.
